# The genome sequence of the zebra danio, *Danio rerio* (Hamilton, 1822) SAT strain (Cypriniformes: Danionidae)

**DOI:** 10.12688/wellcomeopenres.24569.2

**Published:** 2026-03-19

**Authors:** Kerstin Howe, Caroline Howard, Shane A. McCarthy, Jonathan M. D. Wood, Wellcome Sanger Institute Tree of Life Management, Samples and Laboratory team, Wellcome Sanger Institute Scientific Operations: Sequencing Operations, Wellcome Sanger Institute Tree of Life Core Informatics team

**Affiliations:** 1 Wellcome Sanger Institute, Hinxton, England, UK

**Keywords:** Danio rerio, zebra danio, genome sequence, chromosomal, Cypriniformes

## Abstract

We present a genome assembly from a specimen of *Danio rerio* (zebra danio; Chordata; Actinopteri; Cypriniformes; Danionidae). The genome sequence has a total length of 1 413.66 megabases. Most of the assembly (99.85%) is scaffolded into 25 chromosomal pseudomolecules. The mitochondrial genome was also assembled, with a length of 16.6 kilobases.

## Species taxonomy

Eukaryota; Opisthokonta; Metazoa; Eumetazoa; Bilateria; Deuterostomia; Chordata; Craniata; Vertebrata; Gnathostomata; Teleostomi; Euteleostomi; Actinopterygii; Actinopteri; Neopterygii; Teleostei; Osteoglossocephalai; Clupeocephala; Otomorpha; Ostariophysi; Otophysi; Cypriniphysae; Cypriniformes; Cyprinoidei; Danionidae; Danioninae; *Danio*; *Danio rerio* (Hamilton, 1822) (NCBI:txid7955).

## Background

**Figure 1.**
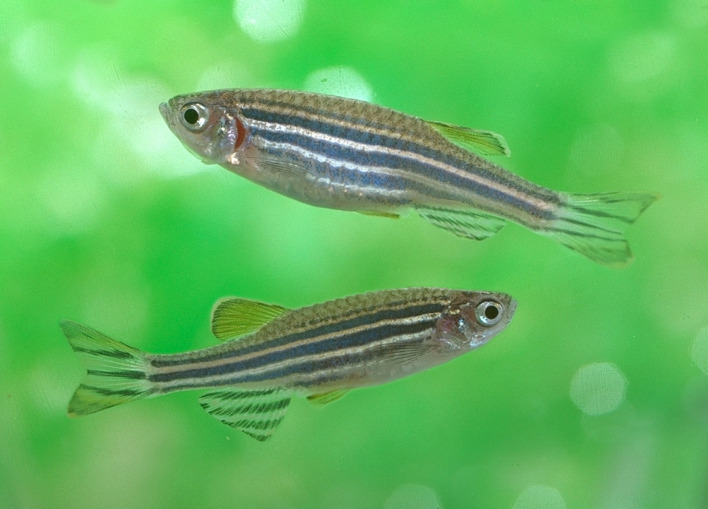
Photograph of *Danio rerio* by Tohru Murakami.

*D. rerio* ( Figure 1), commonly known as the zebrafish, serves as a model organism in various fields of biological research, including genetics, developmental biology, and toxicology ( Lieschke & Currie, 2007). Native to the freshwater habitats of South Asia, the genome of this teleost fish was fully sequenced in 2013 ( Howe *et al.*, 2013). The genome comprises approximately 1.4 gigabases, distributed across 25 chromosomes. Notably, the zebrafish genome exhibits a high degree of synteny with the human genome, making it a valuable tool for studying gene function and regulation ( Howe *et al.*, 2013; Postlethwait *et al.*, 2000).

One of the advantages of using *D. rerio* as a model organism is its rapid embryonic development, which is largely transparent and easily observable under a microscope ( Kimmel *et al.*, 1995). This facilitates real-time analysis of developmental processes. Additionally, the zebrafish is amenable to genetic manipulation, including techniques such as CRISPR/Cas9, which allows for targeted gene editing ( Hwang *et al.*, 2013). Its relatively low maintenance cost and high fecundity further contribute to its utility in research settings ( Lawrence, 2007). The availability of a fully sequenced genome has accelerated functional genomics studies in zebrafish, enabling researchers to perform genome-wide association studies (GWAS), transcriptomics, and other high-throughput analyses ( Lieschke & Currie, 2007). Consequently, *D. rerio* continues to be an indispensable resource in advancing our understanding of vertebrate biology, genetics, and disease mechanisms.

We present a chromosomally complete genome sequence for *D. rerio*, the zebra danio. This genome was assembled using the Tree of Life pipeline from a specimen collected in WSI, Hinxton, East Anglia, United Kingdom.

## Methods

### Sample acquisition

The specimen used for genome sequencing was a laboratory specimen of *D. rerio* SAT strain (specimen ID SAN30000124, ToLID fDanRer4), collected on 2018-05-05 at the Wellcome Sanger Institute, Hinxton, England, UK.

### Nucleic acid extraction

Nucleic acid extraction was carried out using BioNano Prep Cell Culture DNA Isolation Protocol. In this method, the cells are first embedded in agarose to provide structural support during the extraction process. The agarose-embedded cells are then treated with lysis buffers to break down the cell membranes and release the DNA. The process also involves proteinase digestion to remove proteins, followed by a series of washes to purify the DNA.

### Sequencing

Library preparation and sequencing were performed at the WSI Scientific Operations core. Pacific Biosciences (PacBio) HiFi and 10X Genomics read cloud sequencing libraries were constructed according to manufacturers’ instructions. Sequencing was performed by the Scientific Operations core at the Wellcome Sanger Institute on Pacific Biosciences SEQUEL II and Illumina HiSeq X instruments. Hi-C data were generated using the Arima Hi-C kit v2 (Arima Genomics, San Diego, USA) using the Illumina NovaSeq 6000 instrument.

### Genome assembly

Prior to assembly of the PacBio HiFi reads, a database of *k*-mer counts ( *k* = 31) was generated from the filtered reads using FastK. GenomeScope2 ( Ranallo-Benavidez *et al.*, 2020) was used to analyse the *k*-mer frequency distributions, providing estimates of genome size, heterozygosity, and repeat content.

The assembly process included the following sequence of steps: initial PacBio assembly generation with Hifiasm ( Cheng *et al.*, 2021) with the --primary option. Haplotypic duplications were identified and removed using purge_dups ( Guan *et al.*, 2020). This was followed by short-read polishing using FreeBayes-called variants (Garrison *et al.*, 2012) from 10X Genomics Chromium reads aligned with LongRanger, and Hi-C based scaffolding with SALSA2 ( Ghurye *et al.*, 2019). The scaffolded assemblies were evaluated using Gfastats ( Formenti *et al.*, 2022), BUSCO ( Manni *et al.*, 2021) and MERQURY.FK ( Rhie *et al.*, 2020).

The mitochondrial genome was assembled using MitoHiFi ( Uliano-Silva *et al.*, 2023), which runs MitoFinder ( Allio *et al.*, 2020) and uses these annotations to select the final mitochondrial contig and to ensure the general quality of the sequence.

### Assembly curation

Manual curation was conducted primarily in PretextView and HiGlass ( Kerpedjiev *et al.*, 2018). Scaffolds were visually inspected and corrected as described by Howe *et al*. (2021). Manual corrections included 41 breaks, 220 joins, and removal of one haplotypic duplication. We noted that the order and orientation of scaffolds is uncertain for the repetitive regions on chromosome 4 (38.2–55.2 Mbp), chromosome 8 (36.6–40.0 Mbp). Chromosome-scale scaffolds are named by synteny to the *D. rerio* assembly GCA_020184715.1. The curation process is documented at https://gitlab.com/wtsi-grit/rapid-curation . PretextSnapshot was used to generate a Hi-C contact map of the final assembly.

### Assembly quality assessment

The Merqury.FK tool ( Rhie *et al.*, 2020) was run in a Singularity container ( Kurtzer *et al.*, 2017) to evaluate *k*-mer completeness and assembly quality for the primary and alternate haplotypes using the *k*-mer database ( *k* = 31) computed prior to genome assembly. The analysis outputs included assembly QV scores and completeness statistics.

The genome was analysed using the BlobToolKit pipeline, a Nextflow implementation of the earlier Snakemake version ( Challis *et al.*, 2020). The pipeline aligns PacBio reads using minimap2 ( Li, 2018) and SAMtools ( Danecek *et al.*, 2021) to generate coverage tracks. It runs BUSCO ( Manni *et al.*, 2021) using lineages identified by querying the GoaT database ( Challis *et al.*, 2023). For the three domain-level lineages, BUSCO genes are aligned to the UniProt Reference Proteomes database ( Bateman *et al.*, 2023) using DIAMOND blastp ( Buchfink *et al.*, 2021). The genome is divided into chunks based on the density of BUSCO genes from the closest taxonomic lineage, and each chunk is aligned to the UniProt Reference Proteomes database with DIAMOND blastx. Sequences without hits are chunked using seqtk and aligned to the NT database with blastn ( Altschul *et al.*, 1990). The BlobToolKit suite consolidates all outputs into a blobdir for visualisation. The BlobToolKit pipeline was developed using nf-core tooling ( Ewels *et al.*, 2020) and MultiQC ( Ewels *et al.*, 2016), with package management via Conda and Bioconda ( Grüning *et al.*, 2018), and containerisation through Docker ( Merkel, 2014) and Singularity ( Kurtzer *et al.*, 2017).

## Genome sequence report

### Sequence data

**Figure 2.**
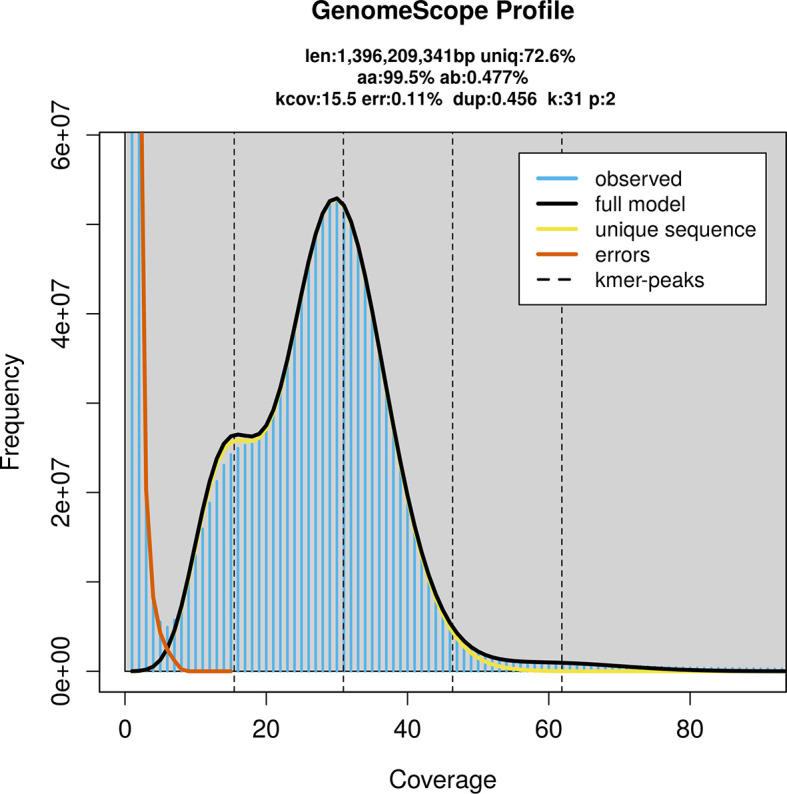
Frequency distribution of *k*-mers generated using GenomeScope2. The plot shows observed and modelled *k*-mer spectra, providing estimates of genome size, heterozygosity, and repeat content based on unassembled sequencing reads.

The genome of a specimen of *D. rerio* was sequenced using Pacific Biosciences single-molecule HiFi long reads, generating 44.85 Gb (gigabases) from 5.52 million reads, which were used to assemble the genome. GenomeScope2.0 analysis estimated the haploid genome size at 1 396.21 Mb, with a heterozygosity of 0.48% and repeat content of 27.43% ( Figure 2). These estimates guided expectations for the assembly. Based on the estimated genome size, the sequencing data provided approximately 31× coverage. Hi-C sequencing produced 145.13 Gb from 961.14 million reads, which were used to scaffold the assembly. Table 1 summarises the specimen and sequencing details.

**Table 1.** Specimen and sequencing data for BioProject PRJEB52651.

Platform	PacBio HiFi	Illumina 10X	Hi-C
**ToLID**	fDanRer4	fDanRer4	fDanRer6
**Specimen ID**	SAN30000124	SAN30000124	SAN30000116
**BioSample (source** **individual)**	SAMEA14088276	SAMEA14088276	SAMEA14088277
**BioSample (tissue)**	SAMEA14088283	SAMEA14088283	SAMEA14088284
**Instrument**	Sequel IIe	Illumina NovaSeq 6000	Illumina NovaSeq 6000
**Run accessions**	ERR9709335–ERR9709337	ERR9710913–ERR9710916	ERR9710907–ERR9710912
**Read count total**	5.52 million	527.58 million	961.14 million
**Base count total**	44.85 Gb	79.67 Gb	145.13 Gb

### Assembly statistics

The primary haplotype was assembled, and contigs corresponding to an alternate haplotype were also deposited in INSDC databases. The final assembly has a total length of 1413.66 Mb in 72 scaffolds, with a scaffold N50 of 58.63 Mb ( Table 2).

**Table 2.** Genome assembly statistics.

**Assembly name**	fDanRer4.1
**Assembly accession**	GCA_944039275.1
**Alternate haplotype** **accession**	GCA_944039255.1
**Assembly level**	chromosome
**Span (Mb)**	1413.66
**Number of chromosomes**	25
**Number of contigs**	453
**Contig N50**	6.93 Mb
**Number of scaffolds**	72
**Scaffold N50**	58.63 Mb
**Organelles**	Mitochondrial genome: 16.6 kb

**Figure 3.**
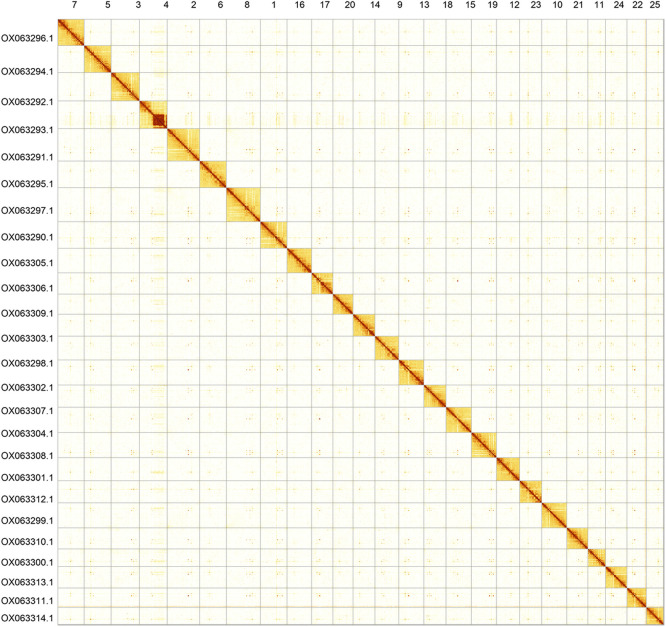
Hi-C contact map of the *Danio rerio* genome assembly. Assembled chromosomes are shown in order of size and labelled along the axes. The plot was generated using PretextSnapshot.

Most of the assembly sequence (99.85%) was assigned to 25 chromosomal-level scaffolds. These chromosome-level scaffolds, confirmed by Hi-C data, are named according to synteny ( Figure 3; Table 3).

**Table 3.** Chromosomal pseudomolecules in the primary genome assembly of *Danio rerio* fDanRer4.

INSDC accession	Molecule	Length (Mb)	GC%
OX063290.1	1	61.81	36.50
OX063291.1	2	63.03	37
OX063292.1	3	66.03	37.50
OX063293.1	4	64.35	38
OX063294.1	5	75.98	37
OX063295.1	6	62.07	36.50
OX063296.1	7	79.03	37.50
OX063297.1	8	62.05	37.50
OX063298.1	9	57.30	36.50
OX063299.1	10	49.66	38
OX063300.1	11	47.03	36.50
OX063301.1	12	50.94	36
OX063302.1	13	55.53	37
OX063303.1	14	58.44	37.50
OX063304.1	15	51.86	37.50
OX063305.1	16	58.78	37.50
OX063306.1	17	58.64	38
OX063307.1	18	53.84	36.50
OX063308.1	19	51.29	36.50
OX063309.1	20	58.63	37
OX063310.1	21	49.14	37
OX063311.1	22	41.12	37.50
OX063312.1	23	49.89	37.50
OX063313.1	24	45.35	37.50
OX063314.1	25	39.74	36.50
OX063315.1	MT	0.02	40

The mitochondrial genome was also assembled. This sequence is included as a contig in the multifasta file of the genome submission and as a standalone record.

### Assembly quality metrics

**Figure 4.**
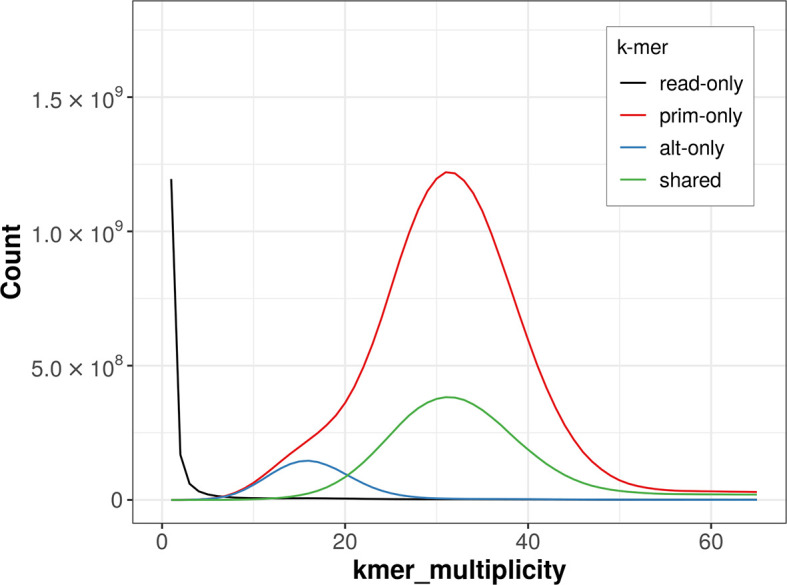
Evaluation of *k*-mer completeness using MerquryFK. This plot illustrates the recovery of *k*-mers from the original read data in the final assemblies. The horizontal axis represents *k*-mer multiplicity, and the vertical axis shows the number of *k*-mers. The black curve represents *k*-mers that appear in the reads but are not assembled. The green curve (the homozygous peak) corresponds to *k*-mers shared by both haplotypes and the red and blue curves (the heterozygous peaks) show *k*-mers found only in one of the haplotypes.

The combined primary and alternate assemblies achieve an estimated QV of 51.8. The *k*-mer completeness is 90% for the primary assembly, 29.91% for the alternate haplotype, and 99.10% for the combined assemblies ( Figure 4). BUSCO v.5.3.2 analysis using the actinopterygii_odb10 reference set ( *n* = 3 640) identified 97.7% of the expected gene set (single = 96.4%, duplicated = 1.2%).

**Figure 5.**
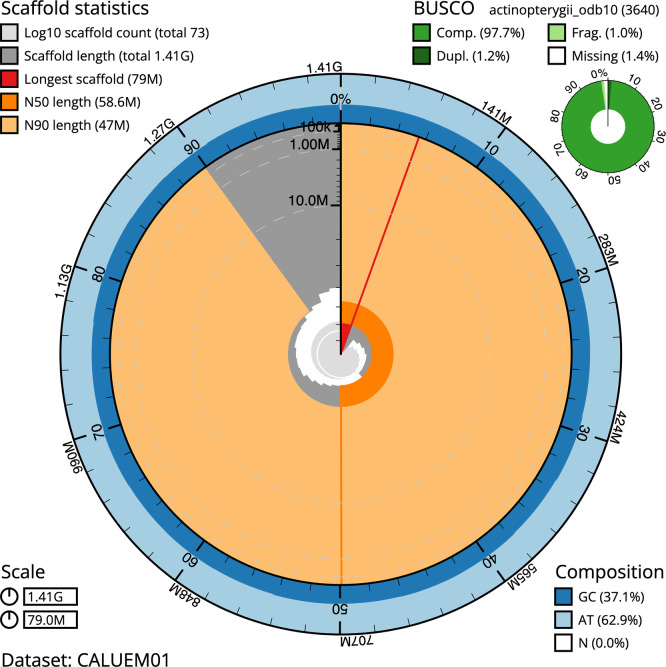
Assembly metrics for fDanRer4.1. The BlobToolKit snail plot provides an overview of assembly metrics and BUSCO gene completeness. The circumference represents the length of the whole genome sequence, and the main plot is divided into 1,000 bins around the circumference. The outermost blue tracks display the distribution of GC, AT, and N percentages across the bins. Scaffolds are arranged clockwise from longest to shortest and are depicted in dark grey. The longest scaffold is indicated by the red arc, and the deeper orange and pale orange arcs represent the N50 and N90 lengths. A light grey spiral at the centre shows the cumulative scaffold count on a logarithmic scale. A summary of complete, fragmented, duplicated, and missing BUSCO genes in the set is presented at the top right. An interactive version of this figure can be accessed on the BlobToolKit viewer.

**Figure 6.**
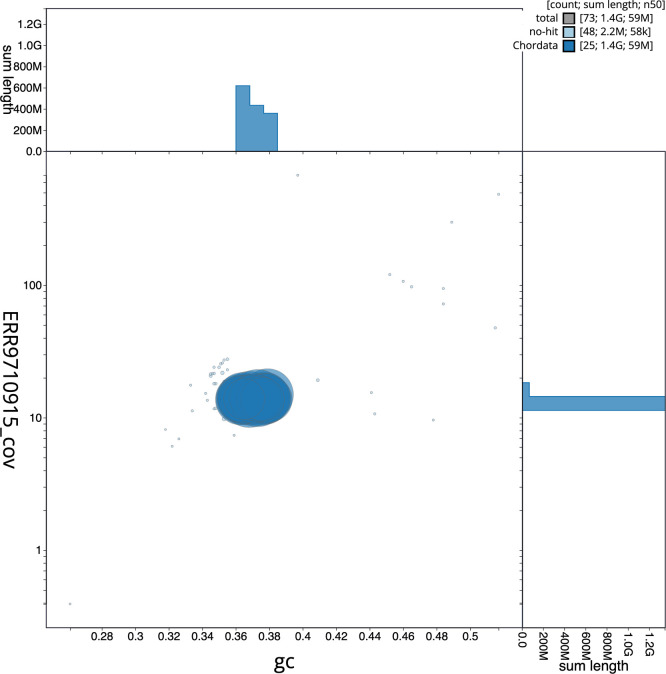
BlobToolKit GC-coverage plot for fDanRer4.1. Blob plot showing sequence coverage (vertical axis) and GC content (horizontal axis). The circles represent scaffolds, with the size proportional to scaffold length and the colour representing phylum membership. The histograms along the axes display the total length of sequences distributed across different levels of coverage and GC content. An interactive version of this figure is available on the BlobToolKit viewer.

The snail plot in Figure 5 summarises the scaffold length distribution and other assembly statistics for the primary assembly. The blob plot in Figure 6 shows the distribution of scaffolds by GC proportion and coverage.

Table 4 lists the assembly metric benchmarks adapted from Rhie *et al.* (2021) the Earth BioGenome Project Report on Assembly Standards September 2024. The EBP metric, calculated for the primary assembly, is **6.C.Q53**, meeting the recommended reference standard.

**Table 4.** Earth Biogenome Project summary metrics for the *Danio rerio* assembly.

Measure	Value	Benchmark
EBP summary (primary)	6.C.Q53	6.C.Q40
Contig N50 length	6.93 Mb	≥ 1 Mb
Scaffold N50 length	58.63 Mb	= chromosome N50
Consensus quality (QV)	Primary: 53.7; alternate: 48.7; combined: 51.8	≥ 40
*k*-mer completeness	Primary: 90.00%; alternate: 29.91%; combined: 99.10%	≥ 95%
BUSCO	C:97.7%[S:96.4%; D:1.2%]; F:1.0%; M:1.4%; n:3 640	S > 90%; D < 5%
Percentage of assembly assigned to chromosomes	99.85%	≥ 90%

## Wellcome Sanger Institute – Legal and Governance

The materials that have contributed to this genome note have been supplied by a Tree of Life collaborator. The Wellcome Sanger Institute employs a process whereby due diligence is carried out proportionate to the nature of the materials themselves, and the circumstances under which they have been/are to be collected and provided for use. The purpose of this is to address and mitigate any potential legal and/or ethical implications of receipt and use of the materials as part of the research project, and to ensure that in doing so, we align with best practice wherever possible. The overarching areas of consideration are:

• Ethical review of provenance and sourcing of the material
• Legality of collection, transfer and use (national and international).

Each transfer of samples is undertaken according to a Research Collaboration Agreement or Material Transfer Agreement entered into by the Tree of Life collaborator, Genome Research Limited (operating as the Wellcome Sanger Institute), and in some circumstances, other Tree of Life collaborators.

## Data availability

European Nucleotide Archive: *D. rerio* (zebrafish). Accession number PRJEB52651. The genome sequence is released openly for reuse. The *D. rerio* genome sequencing initiative is part of the Sanger Institute Tree of Life Programme (PRJEB43745) and Vertebrate Genomes Project (PRJNA489243). All raw sequence data and the assembly have been deposited in INSDC databases. Raw data and assembly accession identifiers are reported in Table 1 and Table 2.

Pipelines used for genome assembly at the WSI Tree of Life are available at https://pipelines.tol.sanger.ac.uk/pipelines. Table 5 lists software versions used in this study.

**Table 5.** Software versions and sources.

Software	Version	Source
BEDTools	2.30.0	https://github.com/arq5x/bedtools2
BLAST	2.14.0	ftp://ftp.ncbi.nlm.nih.gov/blast/executables/blast+/
BlobToolKit	4.3.9	https://github.com/blobtoolkit/blobtoolkit
BUSCO	5.3.2	https://gitlab.com/ezlab/busco
bwa-mem2	2.2.1	https://github.com/bwa-mem2/bwa-mem2
Cooler	0.8.11	https://github.com/open2c/cooler
fasta_windows	0.2.4	https://github.com/tolkit/fasta_windows
FastK	1.1	https://github.com/thegenemyers/FASTK
freebayes	v1.3.1–17-gaa2ace8	https://github.com/freebayes/freebayes
GenomeScope2.0	2.0.1	https://github.com/tbenavi1/genomescope2.0
Gfastats	1.3.6	https://github.com/vgl-hub/gfastats
GoaT CLI	0.2.5	https://github.com/genomehubs/goat-cli
Hifiasm	0.15.3-r339	https://github.com/chhylp123/hifiasm
HiGlass	1.13.4	https://github.com/higlass/higlass
longranger	2.2.2	https://github.com/10XGenomics/longranger
MerquryFK	1.1.2	https://github.com/thegenemyers/MERQURY.FK
MitoHiFi	2	https://github.com/marcelauliano/MitoHiFi
MultiQC	1.14; 1.17 and 1.18	https://github.com/MultiQC/MultiQC
PretextSnapshot	N/A	https://github.com/sanger-tol/PretextSnapshot
PretextView	0.2.5	https://github.com/sanger-tol/PretextView
purge_dups	1.2.3	https://github.com/dfguan/purge_dups
SALSA	2.2	https://github.com/marbl/SALSA
Seqtk	1.3	https://github.com/lh3/seqtk
Singularity	3.9.0	https://github.com/sylabs/singularity

### Author collectives

• Members of the Wellcome Sanger Institute Tree of Life Management, Samples and Laboratory team
• Members of Wellcome Sanger Institute Scientific Operations – Sequencing Operations
• Members of the Wellcome Sanger Institute Tree of Life Core Informatics team
