# Supplementary material for: The genome sequence of the zebra danio, Danio rerio (Hamilton, 1822) SAT strain (Cypriniformes: Danionidae)
Source: Wellcome Open Res. 2026 Mar 19;10:330. Originally published 2025 Jul 4. [Version 2] doi: 10.12688/wellcomeopenres.24569.2 (PMC12405855; doi:10.12688/wellcomeopenres.24569.2)
Supplement: Peer review history

# Reviewer report: Reviewer response for version 2

**DOI:** 10.21956/wellcomeopenres.28832.r151120

**Published:** 2026-04-20

**Authors:** Jayan Duminda M Senevirathna

**Affiliations:** 1 Uva Wellassa University, Badulla, Uva Province, Sri Lanka

In the keywords, scientific name should be italic.

Are sufficient details of methods and materials provided to allow replication by others?

Yes

Is the rationale for creating the dataset(s) clearly described?

Yes

Are the datasets clearly presented in a useable and accessible format?

Yes

Are the protocols appropriate and is the work technically sound?

Yes

Reviewer Expertise:

Aquatic Genetics and Biotechnology

I confirm that I have read this submission and believe that I have an appropriate level of expertise to confirm that it is of an acceptable scientific standard.

# Reviewer report: Reviewer response for version 2

**DOI:** 10.21956/wellcomeopenres.28832.r151119

**Published:** 2026-03-23

**Authors:** Annabel Whibley

**Affiliations:** 1 The University of Auckland, Auckland, New Zealand; 2 Grapevine Improvement, Bragato Research Institute, Lincoln, New Zealand

No further comments.

Are sufficient details of methods and materials provided to allow replication by others?

Yes

Is the rationale for creating the dataset(s) clearly described?

Yes

Are the datasets clearly presented in a useable and accessible format?

Yes

Are the protocols appropriate and is the work technically sound?

Yes

Reviewer Expertise:

Genomics, Bioinformatics, Evolution

I confirm that I have read this submission and believe that I have an appropriate level of expertise to confirm that it is of an acceptable scientific standard.

# Reviewer report: Reviewer response for version 1

**DOI:** 10.21956/wellcomeopenres.27078.r128034

**Published:** 2025-10-15

**Authors:** Shigehiro Kuraku

**Affiliations:** 1 National Institute of Genetics, Shizuoka, Japan

The authors of this manuscript built a new genome assembly of zebrafish Danio rerio and report the basic statistics of the sequences in the assembly. I see the merit of publishing it but suggest improving the manuscript considering the points listed below.

 I could not get which strain/breed of zebrafish was used, AB, Tuebingen, or ...? It is astonishing that the manuscript does not mention it, and 'a laboratory specimen' does not answer this question. 

 'the genome of this teleost fish was fully sequenced in 2013' - I guess 'the genome analysis of this teleost fish' was first reported in publication in 2013' is more correct. What does 'fully' mean here? The genome was sequenced even much earlier, I believe.  

 ’Notably, the zebrafish genome exhibits a high degree of synteny with the human genome .... ' - I do not think the degree is significantly higher than other teleost fishes. I suggest the authors tone down here.

 The abbreviation of WSI appears too suddenly (and later spelled out fully).

 As far as I read the Methods section, I could not get from which tissue the chromatin sample was prepared for Hi-C library preparation, as well as from which tissue genomic DNA was extracted. The authors should state them.

 Table 2 says that the assembly contains 72 scaffolds. Do these 72 include what the authors call chromosomes? It was not clear to me.

 'These chromosome-level scaffolds, confirmed by Hi-C data, are named according to synteny' - I did not get what 'synteny' in this sentence means. Figure 3 does not show what it means. Also, the legend of Figure 3 should guide to readers with what the digits above the figure mean and the IDs on the left mean. It is also important to clearly mention that the authors have maintained the linkage group IDs in the previously released genome assembly, and this is because the IDs are not given according to their sequence lengths, if I understand correctly.

 Most importantly, NCBI now hosts a seemingly complete ('T2T') genome assembly of the same species. I wonder how the assembly version presented in this manuscript under review is related to this putative T2T assembly. What is the advantage of using the assembly version presented in this manuscript under review? It is worth clearly stating any advantage.

Are sufficient details of methods and materials provided to allow replication by others?

No

Is the rationale for creating the dataset(s) clearly described?

Yes

Are the datasets clearly presented in a useable and accessible format?

Yes

Are the protocols appropriate and is the work technically sound?

Yes

Reviewer Expertise:

NA

I confirm that I have read this submission and believe that I have an appropriate level of expertise to confirm that it is of an acceptable scientific standard, however I have significant reservations, as outlined above.

# Reviewer report: Reviewer response for version 1

**DOI:** 10.21956/wellcomeopenres.27078.r127660

**Published:** 2025-09-18

**Authors:** Luohao Xu

**Affiliations:** 1 MOE Key Laboratory of Freshwater Fish Reproduction and Development, School of Life Sciences, Southwest University, Chongqing, China

Zebrafish in one of the most important model animals, but a reference genome has not been available until recently. In this study, a new genome assembly for zebrafish is presented using HiFi and Hi-C sequencing data. The quality meets the VGP standards, but the assembly for the heterochramatic region (reference 1) may have not be well resolved. It is also worth mentioning that a T2T genome assembly for zebrafish seems to be available ( https://www.ncbi.nlm.nih.gov/datasets/genome/GCF_049306965.1/). The difference is likely that ONT ultra-long reads have not been used in this study.   

 What is the sex of the sample?

 The hifiasm version used in this study is a bit outdated.

Are sufficient details of methods and materials provided to allow replication by others?

Yes

Is the rationale for creating the dataset(s) clearly described?

Yes

Are the datasets clearly presented in a useable and accessible format?

Yes

Are the protocols appropriate and is the work technically sound?

Yes

Reviewer Expertise:

Genomics

I confirm that I have read this submission and believe that I have an appropriate level of expertise to confirm that it is of an acceptable scientific standard.

## References

[1] : A map of cis-regulatory elements and 3D genome structures in zebrafish. *Nature* .2020;588(7837) : 10.1038/s41586-020-2962-9 337-343 10.1038/s41586-020-2962-9 PMC818357433239788

# Reviewer report: Reviewer response for version 1

**DOI:** 10.21956/wellcomeopenres.27078.r128033

**Published:** 2025-09-02

**Authors:** Annabel Whibley

**Affiliations:** 1 The University of Auckland, Auckland, New Zealand; 2 Grapevine Improvement, Bragato Research Institute, Lincoln, New Zealand

Howe and colleagues report the genome assembly and annotation of the zebrafish (or zebra danio, *Danio rerio*) using Darwin Tree of Life protocols and pipelines. The recovered assembly is of high quality (formally 6.C.Q53) with nearly 99.9% of the 72 scaffolds assigned to one of the 25 chromosomes. The report follows the standard template- methods are current and appropriate, the protocols and sample metadata are comprehensively documented in a well-structured report. This will prove a valuable resource for the scientific community.

 As an important biological model and early adopter of genomics technologies, I would have liked to learn more about how this genome assembly differs from other references generated for this species and to what extent this version might provide improved resolution. I am also curious about the patterns shown in the chromosome 4 contact map and what might be driving that.  I accept that these are questions that fall beyond the scope of the Data Note, which prioritizes standardized reporting and quick release of datasets to the community. But I do look forward to seeing more comparative analysis of this assembly in the future.

Are sufficient details of methods and materials provided to allow replication by others?

Yes

Is the rationale for creating the dataset(s) clearly described?

Yes

Are the datasets clearly presented in a useable and accessible format?

Yes

Are the protocols appropriate and is the work technically sound?

Yes

Reviewer Expertise:

Genomics, Bioinformatics, Evolution

I confirm that I have read this submission and believe that I have an appropriate level of expertise to confirm that it is of an acceptable scientific standard.

# Reviewer report: Reviewer response for version 1

**DOI:** 10.21956/wellcomeopenres.27078.r128039

**Published:** 2025-09-01

**Authors:** Jayan Duminda M Senevirathna

**Affiliations:** 1 Uva Wellassa University, Badulla, Uva Province, Sri Lanka

The written format of the article is acceptable. There appears to be a space before the last three digits of certain numerical values, which may cause readability issues. Replace spaces with commas in accordance with standard numerical formatting (e.g., 1,396.21 Mb instead of 1 396.21 Mb).

 Although the genome assembly completeness for chromosome scaffolding is reported as 99.85%, the term *pseudochromosomes* is still used. Please clarify the rationale for employing this terminology despite the high assembly completeness.

Are sufficient details of methods and materials provided to allow replication by others?

Yes

Is the rationale for creating the dataset(s) clearly described?

Yes

Are the datasets clearly presented in a useable and accessible format?

Yes

Are the protocols appropriate and is the work technically sound?

Yes

Reviewer Expertise:

Aquatic Genetics and Biotechnology

I confirm that I have read this submission and believe that I have an appropriate level of expertise to confirm that it is of an acceptable scientific standard.
